# Reversible ST‐T Segment Changes Induced by Anamorelin: A Case Report

**DOI:** 10.1002/rcr2.70398

**Published:** 2025-11-05

**Authors:** Hayato Akaishi, Tomonori Makiguchi, Yuji Ishida, Takuya Shimanaka, Hisashi Tanaka, Kageaki Taima, Sadatomo Tasaka

**Affiliations:** ^1^ Department of Respiratory Medicine Hirosaki University Graduate School of Medicine Aomori Japan; ^2^ Department of Cardiology and Nephrology Hirosaki University Graduate School of Medicine Aomori Japan

**Keywords:** anamorelin, cachexia, calcium channel L‐type receptors, CYP3A4, ST‐segment changes

## Abstract

Anamorelin has been approved for cancer cachexia in Japan. Anamorelin has been known to cause adverse events including QT prolongation and can lead to fatal arrhythmia. We report a first case of a male patient with non‐small cell lung cancer who showed ST‐T segment change following anamorelin. Five days after starting anamorelin, ST‐segment elevation in lateral leads and reciprocal ST depression in the inferior leads were noted. He did not complain about chest pain. Neither echocardiography nor computed tomography angiography revealed abnormal findings. Anamorelin was immediately discontinued. ST‐T segment change recovered gradually and returned to normal on Day 19. During the course, cardiac enzymes had never been elevated. The manufacturer reports that anamorelin is shown to weakly but competitively bind to calcium channel L‐type receptors, which could inhibit repolarization. Even if patients do not complain of chest discomfort, an early ECG should be considered.

## Graphical abstract

Anamorelin, which has been approved for cancer cachexia in Japan, has been known to cause adverse events including QT prolongation and can lead to fatal arrhythmia. We report the first case of a male patient with non‐small cell lung cancer who showed ST‐T segment change following anamorelin. He did not complain about chest pain and tests were normal. The treatment was discontinued and ST‐T segment change recovered gradually and returned to normal. Even if patients do not complain of chest discomfort, an early ECG should be considered.

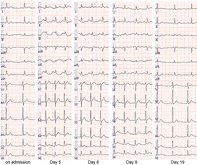

## 1 Introduction

Cancer cachexia is a catabolic phenomenon characterised by anorexia and reduced skeletal muscle [1]. This condition leads to physical inactivity, low quality of life, poor tolerance to anticancer treatment. Anamorelin, which is an orally active, synthetic mimetic of ghrelin, is a selective ghrelin‐receptor agonist. Because anamorelin improved lean body mass, body weight and appetite in Japanese patients with non‐small cell lung cancer (NSCLC), it was approved in Japan in 2021 [2]. In prospective post‐marketing surveillance in Japan, treatment‐related adverse events occurred in 15.7% of the patients with NSCLC [3]. Of these, 1.6% (grade ≥ 3: 0.4%) had conduction disorders. Although QT prolongation was reported, ST‐T segment change has never been reported so far.

## 2 Case Report

**FIGURE 1:**
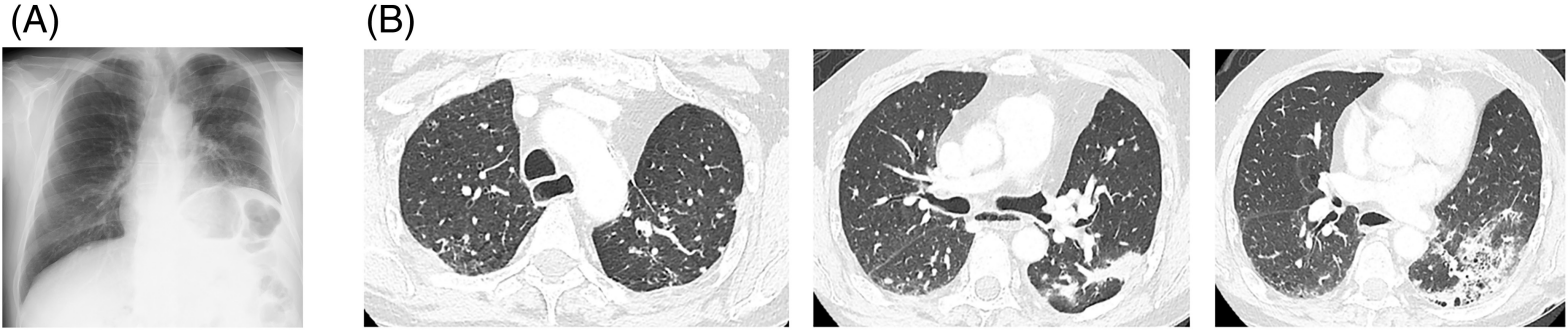
Chest x‐ray and computed tomography before anamorelin. (A) Chest x‐ray showed volume reduction in the left lung due to a previous left upper lobectomy. (B) Chest computed tomography revealed consolidation in the left lower lobe along with bilateral multiple pulmonary metastases.

**FIGURE 2:**
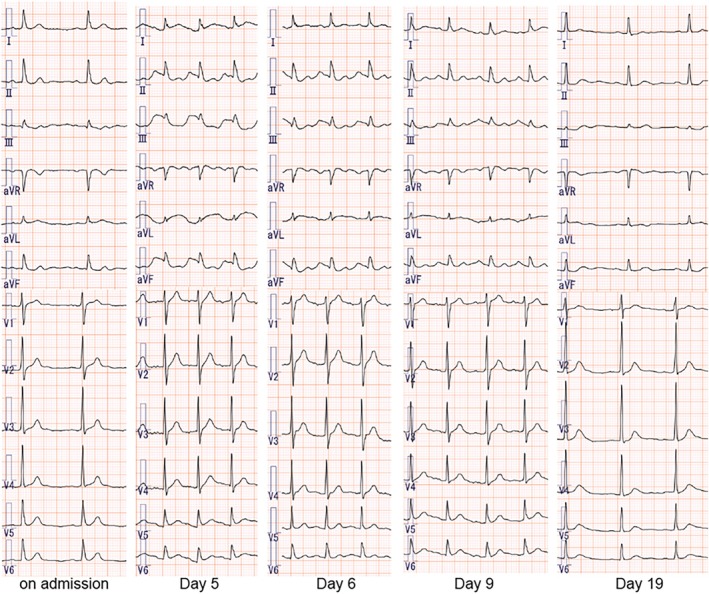
The change of ECG before and after receiving anamorelin. On admission, there are no abnormal findings including ST changes. On Day 5, ST‐T segment elevation in lateral leads (I, aVL, V5, and V6) and reciprocal ST depression in the inferior leads (II, III, and aVF), and tachycardia were noted. Anamorelin was immediately discontinued. On the following day, ST‐T segment changes sustained. On Day 9, ST‐T segment changes recovered to baseline levels. On Day 19, tachycardia had also resolved.

A 65‐year‐old man with NSCLC was admitted to our department to receive chemotherapy. He had no comorbidities other than hypertension, which was treated with azelnidipine. He had a smoking history of 34 pack‐years. He underwent left upper lobectomy 8 years ago and was diagnosed with invasive mucinous adenocarcinoma (pT3N2M0 Stage IIIA). One year later, he experienced recurrence, and the first‐line chemotherapy with afatinib was introduced. As the second‐line treatment, he had received a combination therapy of carboplatin, paclitaxel, bevacizumab and atezolizumab. At this time, he was to receive the first cycle of docetaxel and ramucirumab (DTX + RAM) as the third‐line setting. His vital signs were normal, and no abnormal sounds were noted on auscultation. Chest x‐ray demonstrated a decrease in the volume of the left lung due to the previous left upper lobectomy (Figure 1A). Chest computed tomography revealed the left lower lobe consolidation suggestive of the infiltration of invasive mucinous adenocarcinoma, along with bilateral multiple pulmonary metastases (Figure 1B). No cancer infiltration into the heart or pericardial effusion was observed. The tumour burden was localised to the lungs, and no extrathoracic metastases were present. Because he had lost 4 kg of body weight over the past month, indicating the coexisting cancer cachexia, he started on anamorelin concurrently. ECG on admission showed normal rhythm and no ST‐T segment changes. Five days later, ST‐segment elevation in lateral leads (I, aVL, V5 and V6) and reciprocal ST depression in the inferior leads (II, III and aVF) were noted (Figure 2). Although he had no chest pain, he had tachycardia with a heart rate of 123 bpm. In the laboratory tests, serum sodium and potassium concentrations were 136 and 4.7 mEq/L, respectively (Table 1). CK‐MB and troponin T were not elevated. Similarly, other electrolytes such as potassium, magnesium, and calcium were also normal. Echocardiography and coronary computed tomography angiography revealed no abnormalities. Anamorelin and azelnidipine were immediately discontinued. On the following day (Day 6), the ST‐segment change persisted without elevation in cardiac enzyme levels. On Day 9, the ST‐segment almost recovered to baseline. On Day 19, the tachycardia had also resolved. After the second dose of DTX + RAM, ST‐segment changes did not appear, indicating that the ECG changes were not caused by chemotherapy.

**TABLE 1:** Laboratory findings obtained 5 days later after starting on anamorelin.

Haematology		Chemistry		Serology	
WBC	9420 /μL	TP	7.0 g/dL	Tropnin T	0.008 ng/mL
RBC	10 × 10^4^/μL	Alb	3.2 g/dL	NT‐proBNP	11 pg/mL
Hb	12.9 g/dL	Na	136 mmol/L		
Plt	23.1 × 10^4^/ μL	K	4.7 mmol/L		
		Cl	103 mmol/L		
		Ca	9.5 mg/dL		
		Mg	1.8 mg/dL		
		AST	27 U/L		
		ALT	27 U/L		
		LDH	248 U/L		
		CPK	44 U/L		
		CK‐MB	< 4 U/L		
		BUN	12 mg/dL		
		Cr	0.87 mg/dL		
		CRP	8.0 mg/dL		

## 3 Discussion

While the Japanese post‐marketing surveillance showed conduction disorders in 1.6% of the patients with NSCLC receiving anamorelin, an ST‐segment change has never been reported [3]. In our patient, although the ST‐segment change persisted for more than a week, there were neither elevated cardiac enzymes, nor chest pain, clearly indicating that it is distinct from ischemic heart diseases and vasospastic angina. Generally, long‐standing vasospastic angina, as observed in our patient, leads to an elevation in serum myocardial enzyme levels. Similarly, since giant negative T waves did not appear after the improvement of ST‐segment changes, the condition did not meet the diagnostic criteria for Takotsubo cardiomyopathy. During the preclinical evaluation, anamorelin was screened against 100 receptors as well as binding affinity with the ghrelin receptor [4]. Although detailed data was not provided, the pharmacologic profile indicated that 10 μM anamorelin exhibited competitive binding to the L‐type calcium channel receptors. Generally, calcium channels are involved with repolarization in the myocardium. Yoshitaka et al. reported a case of Brugada syndrome that showed ST‐segment elevation aggravated by a calcium channel blocker [5]. They insisted that attention should be paid when prescribing a calcium channel blocker for the patient with Brugada syndrome. As is the case with this, anamorelin may cause ST‐segment elevation in a specific situation. For instance, when taking it simultaneously with a CYP3A4 inhibitor, which interrupts the metabolism of anamorelin. Although he had not been taking a CYP3A4 inhibitor, he had been taking azelnidipine, a calcium channel blocker, that is also metabolized by CYP3A4. We assume that azelnidipine and anamorelin competitively inhibit the effect of CYP3A4, which may result in amplifying the calcium channel blockade. We should be more careful when administering anamorelin with other drugs metabolized by CYP3A4. Besides the interaction of multiple drugs, the patient condition, including cachexia or renal dysfunction, should be noted as well. In this patient, no conditions that could affect drug metabolism, such as severe liver metastases or liver cirrhosis, were observed. On the other hand, considering the inhibitory effect of ramucirumab on VEGF, ramucirumab has the potential to cause ST‐T segment change. In the REGARD trial, which evaluated the efficacy and safety of ramucirumab monotherapy in those with gastric cancer or gastrooesophageal junction adenocarcinoma who had experienced disease progression following prior chemotherapy, one patient (0.4%) receiving ramucirumab developed Grade 5 acute myocardial infarction. Based on this finding, the manufacturer's instruction raises awareness regarding arterial thromboembolic events including ischemic heart diseases. However, our patient did not show ST‐T segment change after the second dose of DTX + RAM. In addition, there have been no reports regarding the interaction between ramucirumab and calcium channel blockers.

To the best of our knowledge, this is the first reported case demonstrating reversible ST‐segment change following anamorelin. Although the precise mechanism remains unknown, clinicians should consider close ECG monitoring on starting anamorelin, especially when the patient is taking a calcium channel blocker or other drug metabolized by CYP3A4.

## Author Contributions

**Hayato Akaishi:** investigation, writing, review and editing. **Tomonori Makiguchi:** conceptualization, investigation, writing, review and editing. **Yuji, Ishida, Takuya Shimanaka**, **Hisashi Tanaka, Kageaki Taima:** investigation, review and editing. **Sadatomo Tasaka:** investigation, review, editing and supervision.

## Consent

The authors declare that written informed consent was obtained for the publication of this manuscript and accompanying images using the consent form by the Journal.

## Conflicts of Interest

The authors declare no conflicts of interest.

## Data Availability

The data that support the findings of this study are available on request from the corresponding author. The data are not publicly available due to privacy or ethical restrictions.
